# Implementing an evidence-based computerized decision support system linked to electronic health records to improve care for cancer patients: the ONCO-CODES study protocol for a randomized controlled trial

**DOI:** 10.1186/s13012-016-0514-3

**Published:** 2016-11-25

**Authors:** Lorenzo Moja, Alessandro Passardi, Matteo Capobussi, Rita Banzi, Francesca Ruggiero, Koren Kwag, Elisa Giulia Liberati, Massimo Mangia, Ilkka Kunnamo, Michela Cinquini, Roberto Vespignani, Americo Colamartini, Valentina Di Iorio, Ilaria Massa, Marien González-Lorenzo, Lorenzo Bertizzolo, Peter Nyberg, Jeremy Grimshaw, Stefanos Bonovas, Oriana Nanni

**Affiliations:** 1 Department of Biomedical Sciences for Health, University of Milan, Via Pascal 36, 20133 Milan, Italy; 2 Clinical Epidemiology Unit, IRCCS Orthopedic Institute Galeazzi, Via Galeazzi 4, 20161 Milan, Italy; 3 Medical Oncology Unit, IRST Istituto Scientifico Romagnolo per lo Studio e la Cura dei Tumori IRCCS, Meldola, Italy; 4 School of Specialization in Hygiene and Preventive Medicine, University of Milan, Milan, Italy; 5 IRCCS Mario Negri Institute for Pharmacological Research, Via La Masa 19, 20156 Milan, Italy; 6 Cambridge Centre for Health Services Research (CCHSR), Department of Public Health and Primary Care, Cambridge Institute of Public Health, Forvie Site, Robinson Way, Cambridge, CB2 0SR UK; 7 Medilogy Srl, Viale Monza 133, 20125 Milan, Italy; 8 Duodecim Medical Publications Ltd, Kaivokatu 10 A, 00101 Helsinki, Finland; 9 Clinical Epidemiology Program, Ottawa Hospital Research Institute and Department of Medicine, University of Ottawa, 501 Smyth Road, Ottawa, ON K1H 8 L6 Canada; 10 Humanitas Clinical and Research Center, Via Manzoni 56, 20089 Rozzano, Milan Italy

**Keywords:** Computerized decision support systems, Electronic health records, Electronic patient records, Evidence-based medicine, Pragmatic trial, Randomized controlled trial, Reminder systems, Oncology

## Abstract

**Background:**

Computerized decision support systems (CDSSs) are computer programs that provide doctors with person-specific, actionable recommendations, or management options that are intelligently filtered or presented at appropriate times to enhance health care. CDSSs might be integrated with patient electronic health records (EHRs) and evidence-based knowledge.

**Methods/Design:**

The Computerized DEcision Support in ONCOlogy (ONCO-CODES) trial is a pragmatic, parallel group, randomized controlled study with 1:1 allocation ratio. The trial is designed to evaluate the effectiveness on clinical practice and quality of care of a multi-specialty collection of patient-specific reminders generated by a CDSS in the IRCCS Istituto Scientifico Romagnolo per lo Studio e la Cura dei Tumori (IRST) hospital. We hypothesize that the intervention can increase clinician adherence to guidelines and, eventually, improve the quality of care offered to cancer patients. The primary outcome is the rate at which the issues reported by the reminders are resolved, aggregating specialty and primary care reminders. We will include all the patients admitted to hospital services. All analyses will follow the intention-to-treat principle.

**Discussion:**

The results of our study will contribute to the current understanding of the effectiveness of CDSSs in cancer hospitals, thereby informing healthcare policy about the potential role of CDSS use. Furthermore, the study will inform whether CDSS may facilitate the integration of primary care in cancer settings, known to be usually limited. The increasing use of and familiarity with advanced technology among new generations of physicians may support integrated approaches to be tested in pragmatic studies determining the optimal interface between primary and oncology care.

**Trial registration:**

ClinicalTrials.gov, NCT02645357

## Background

### Background and rationale

Most hospitals collect huge amounts of electronic administrative and clinical data. Without returning these data to clinicians, collected e-information about healthcare services fails to inform clinicians [1]. Administrative and demographic information, diagnoses, treatments, prescription drugs, laboratory tests, hospitalizations, and patient insurances, are cumulated. Doctors are primarily involved in the collection of clinical data. Despite doctors invest energies and time collecting patient information during visits, this information, at best, comes back after some times (e.g., at the end of the year), in a report aggregating data from different patients and doctors. So, the feedback is not pertinent to a specific patient (i.e., non-selective) but applies to an average patient visited in the past (i.e. asynchronous) being not that helpful [2]. Computer applications that regularly and effortlessly track key clinical and administrative data and select the information that applies to a single patient may support real-time clinical decision-making conveying on time messages.

One of the major innovations in this field is computerized decision support systems (CDSSs) that are fully integrated with electronic health records (EHRs) and evidence-based knowledge [3]. CDSSs are information technology-based software that provides clinicians, staff, patients, or other individuals with person-specific, actionable recommendations, or management options that are intelligently filtered or presented at appropriate times to enhance health and health care [4, 5].

Current research investigates CDSSs’ potential to assist with problems raised in clinical practice, decrease the rate of medication errors, increase the adherence of clinicians to guideline- or protocol-based care, and, ultimately, to improve the overall efficiency and quality of healthcare delivery systems [6–19].

Few randomized controlled trials (RCTs) have evaluated the impact of CDSSs on health care [5]. Most CDSSs supported few specific clinical decisions restricted to specialized care (e.g., vital parameter monitoring and critical care in intensive care units).

The opportunity to improve patient care, by making medical knowledge readily available to physicians at the point of care, represents one of the main incentives for investing on development and evaluation of these sophisticated information systems.

Despite evidence shows that primary care helps prevent illness and death, regardless of whether the care is supplied in primary or secondary care settings, there is limited research on integrating primary and oncology care [20, 21]. Cancer patients are often old, have comorbidities, and face long diseases and treatments. They would benefit from greater supply of primary care when cared for conditions other than primary [22]. Few CDSSs commercially available are capable to support a wide range of medical conditions and support decisions such as drugs and diagnostic test ordering, encompassing oncology, other specialties and primary care. These tools can potentially support the provision of integrated healthcare services by hospital doctors who are primarily accountable for addressing specialty diseases (i.e., cancer) and developing a continuum between primary and specialty care.

### Objective

Our study aims to evaluate the effectiveness of patient-specific, point-of-care reminders generated by the Medilogy Decision Support System (MediDSS) [23] on clinical practice and on the improvement of quality of care in cancer patients. We hypothesize that the intervention can increase clinician adherence to recommendations targeting decisions both in primary and secondary settings. Adherence to international guidelines, eventually, can improve the quality of care offered to cancer patients and survivors.

## Methods/Design

This protocol is reported in accordance with the SPIRIT 2013 guidance for content of clinical trial protocols [24, 25].

### Trial design

**Fig. 1:**
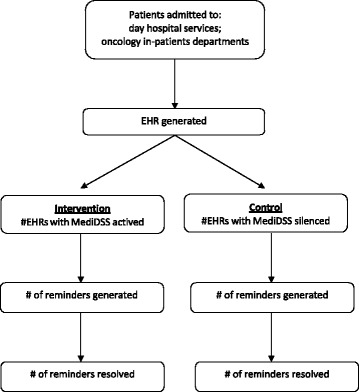
Trial flow chart

The Computerized DEcision Support in ONCOlogy (ONCO-CODES) trial is a pragmatic, parallel group, randomized controlled study with 1:1 allocation ratio. The flow diagram of the study can be found in Fig. 1.

### Study setting

The study will involve the medical staff of the day hospital service and of the oncologic medicine departments of the IRST Istituto Scientifico Romagnolo per lo Studio e la Cura dei Tumori IRCCS (thereafter indicated as IRST), which is a research center specialized in innovative care in the oncologic field and operational since 2007. The hospital is placed in the Emilia-Romagna region, in Italy, has 36 bed places, and is the hub of several “spoke” ambulatory services. The catchment population of the whole network is about 1,130,000 people, while the high specialization level of the hospital service attracts patients from all Northern Italy [26].

The IRST counts more than 4900 admissions per year. In addition to the in-patient wards which admit 1600 patients per year, the day hospital service performs over 3300 admissions annually.

The IRST has a medical staff of 140 doctors for a total of 500 health professionals. Since 2007, the hospital has been electronically tracking all clinical and administrative information through an EHR system based on the “Cartella clinica oncologica” platform (developed by a local software house, Log80) [27].

### Eligibility criteria and recruitment

As a pragmatic clinical trial [28, 29], ONCO-CODES seeks to investigate the effectiveness of the computerized reminders in everyday clinical practice with diverse patients and varying conditions. Thus, we will enroll all patients admitted to the oncology in-patient departments and the day hospital services of the IRST during the study period, without applying any exclusion criteria.

### Intervention

We selected the Medilogy Decision Support System (MediDSS) after a comparative assessment of available editorial products using a predefined set of essential criteria [30, 31]. MediDSS is a product by Medilogy, an Italian developer of scientific software and medical technology. Medilogy translated and adapted Evidence-based medicine electronic decision support (EBMeDS) [32], a CDSS developed by Duodecim Medical Publications Ltd., a company owned by the Finnish Medical Society Duodecim. EBMeDS can be described as a set of rules (scripts) based on EBM guidelines and applied to structured health data. MediDSS further includes knowledge from Swedish, Finnish, INteraction X-referencing (SFINX), a drug-drug interaction database containing concise evidence-based information about the harms and benefits of about 18,000 drug interactions and adverse events [33].

MediDSS may be used as stand-alone application or may integrate structured patient data from EHR to generate patient-specific reminders, therapeutic suggestions, and diagnosis-specific links to full-text guidelines. Reminders are automatically generated and displayed on the monitors of clinicians when they open a patient’s EHR, enter a new diagnosis, prescribe a drug, or when new laboratory test results are available. Reminders were formed using international evidence-based guidelines and subsequently approved by an international panel of experts.

Our study will use international reminders (*n* = 262), which cover a large number of health conditions across specialties and are derived from the EBMeDS, and local reminders (*n* = 39), a series of tailored messages designed by the hospital staff. Moreover, the CDSS provides information about more than 10,000 drug–drug interactions or other drug-related problems, giving advice on how to handle them [34]. For instance, in a diabetic patient, a diagnosis of renal failure, or a laboratory result of creatinine increase, can trigger an alert message to reduce the drug dosage (e.g., cisplatin) based on the patient’s glomerular filtration rate (GFR).There are also several reminders that may help oncologists in the holistic care of the patient. Examples are: starting and maintaining bisphosphonate therapy when a myeloma or metastatic cancer is in the patient’s diagnosis list; alert on platelets monitoring in patients who have recently started treatment with heparins.

The reminders system will be “activated” in the EHR of patients allocated to the intervention group, while the system will be “silenced” in the EHR of those allocated to the control group. Since during care of control group patients, the generated reminders will not be shown to the physicians, control is the standard clinical practice without reminders in use. However, the best evidence for usual care will be available to the physicians at all times during the trial, if the doctor wishes to actively search for clinical practice guidelines or other information sources. All participating physicians will be informed on the availability and use of the MediDSS system.

### Stepped wedge implementation

The intervention is a new technology: its integration in the current hospital system requires the configuration and customization of the software. To allow security controls and successful implementation, the CDSS will be sequentially rolled out to participants over a number of time periods. We anticipate that the number of periods will be limited (i.e., two or three periods). Over an initial period, all participants will receive the intervention. The order in which participants will receive the intervention is not determined at random, but will be determined by selecting physicians prone to provide constructive feedback to the implementation team. The RCT adopts a stepped wedge implementation of the intervention, but not a stepped wedge design [35]. Sequential roll-out of the intervention will not be considered a pilot phase of the trial, but a part of the whole RCT.

### Selection and development of local reminders

In order to encourage the participation of the hospital staff within the study, we invited hospital representatives to assess the priority needs of the hospital wards. Following consultation, an IRST-specific set of reminders has been implemented.

Due to the increasing costs for the national health system of certain kinds of drugs, i.e., high-cost monoclonal antibodies, the Italian Medicines Agency developed managed entry agreements based on a risk-sharing policy. According to it, innovative oncology drugs are the subjects of risk-sharing schemes [36]. The agreements require manufacturers to pay-back part of the price of the drug for each patient who fails to respond to the new treatment. Hospitals track patient eligibility and monitor the use of drugs included in the financial scheme and their outcomes. Evidence collection of treatment completion or interruption owing to progression or toxicity has to follow rigid scheme and timeline. The hospital then applies to the national health system for pay-back. Late request for risk sharing or inaccurate monitoring of progression lead to partial losses in the reimbursement to the hospital, with negative financial impact. To support efficient documentation of treatment failures, a set of reminders was developed to optimize the timing of clinical-instrumental evaluations in accordance with the procedures provided by the risk-sharing schedule.

The local hospital expert group identified the following drugs as of special interest: abiraterone, afatinib, aflibercept, axitinib, azacitidine, bevacizumab, bortezomib, bosutinib, cetuximab, crizotinib, dabrafenib, dasatinib, decitabine, enzalutamide, eribulin, erlotinib, everolimus, gefitinib, ipilimumab, lapatinib, lenalidomide, nilotinib, ofatumumab, paclitaxel, panitumumab, pazopanib, pemetrexed, pertuzumab, plerixafor, ponatinib, sorafenib, sunitinib, temserolimus, trabectidine, trastuzumab, vemurafenib, vinflunina, and vismodegib. The IRST-specific set of reminders was developed taking into consideration the conditions that have to be met for drug full reimbursement, including the schedule for the diagnostic and laboratory analyses required from the Italian Medicines Agency to monitor the drug’s efficacy and safety [37].

In case of changes or updates in the agreements, the reminders will be modified accordingly, in order to guarantee the CDSS reminders to be always up-to-date.

An example of an algorithm is provided in Table 1.

**Table 1:** Algorithm for the correct prescription and the reimbursement by the national health system of a monoclonal antibody

Bevacizumab, an anti-angiogenetic drug, can be prescribed as a first line treatment for non-small cell lung cancer patients. The patients have to be evaluated by imaging tests after the 3rd administration and before the 4th [37]. When the disease progresses, the drug is considered to be not effective: the treatment is discontinued and the hospital is reimbursed for the first three administrations. When the treatment provides the desired results, the hospital can continue and the national health system covers all the costs. In any other case, i.e., the treatment is ineffective but the patient’s evaluation is performed after the 4th administration or the treatment is continued after progression, the hospital will pay for the drugs and get no reimbursement.	

**Fig. 2:**
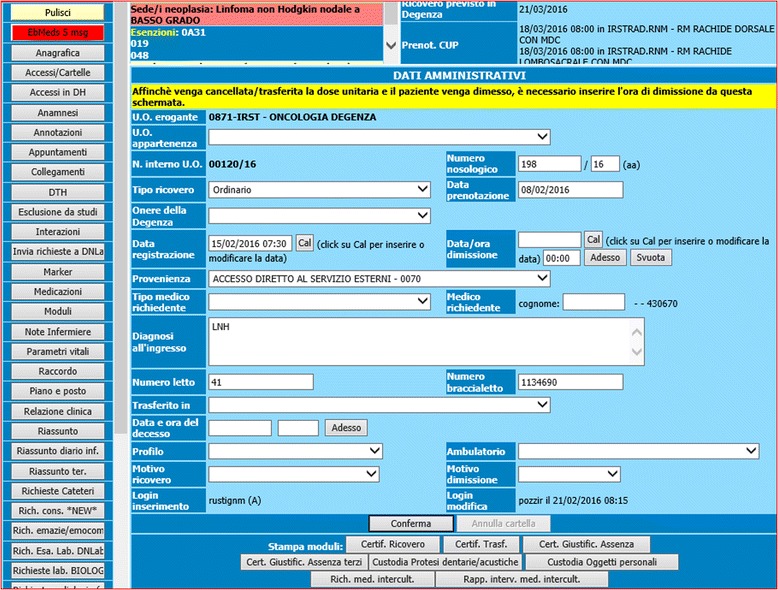
Activation button

**Fig. 3:**
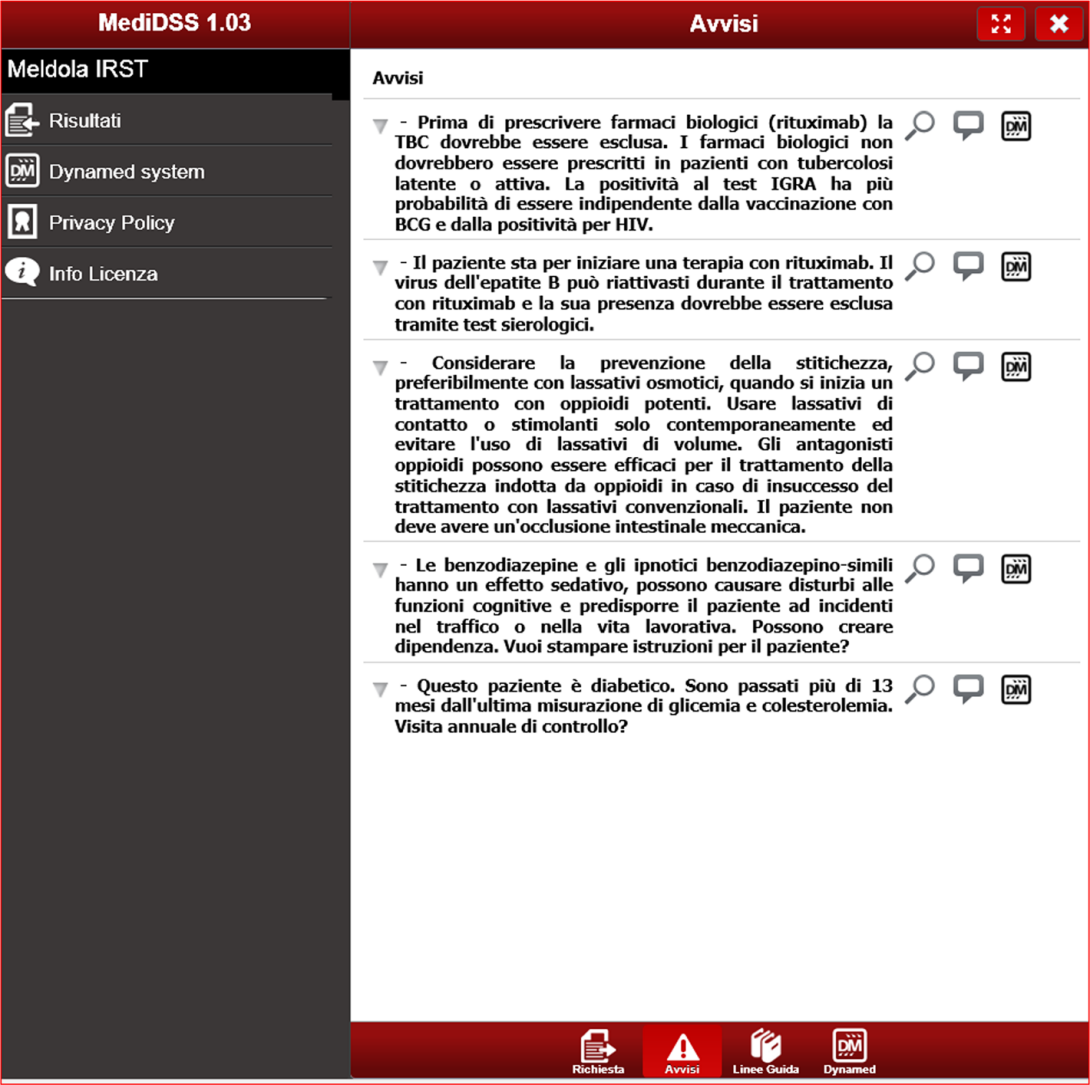
Reminder

Figures 2 and 3 show a snapshot of the activation button and of the actual reminders.

### Qualitative integration

The implementation of the intervention is a key aspect of this RCT: the results will be valid if physicians will use MediDSS in their clinical activities. Healthcare service studies on CDSSs, however, suggested that the mere provision of such technology does not guarantee its uptake. In fact, even if a CDSS is readily available within a hospital, there is a strong tendency to ignore its recommendations, not trusting the majority of its alerts [38]. Our RCT is informed by qualitative interviews aimed to detect how the CDSS fits in into clinical practice by diverse health professionals involved in oncology patient care. The interviews are a part of a larger cross-sectional study, which involves three Italian hospitals [39]. The interviews will explore barriers and facilitators that may hinder the use of a CDSS in specialty settings, including technical (e.g., poor usability or knowledge of system), individual (e.g., negative perception of CDSS or EBM, lack of motivation), group or organizational (e.g., structural or administrative constraints), and cultural factors (e.g., adverse social norms).

The qualitative assessment will inform the trial intervention, enabling possible improvements in the integrity of the intervention and associated compliance. We will collect feedbacks about usability, possible errors, or inaccuracies of the information and recommendations provided. We will offer the best possible solutions to clinicians and hospital staff to overcome these problems, modifying the CDSS software if required and possible. We cannot anticipate the types and level of changes that will be proposed to improve the technology. Physicians will volunteer for interviews, so it is possible we will likely to include disproportionately more of those with strong feelings or requests about the system.

### Study outcomes

Active reminders represent potential problems in patient management: the fewer the number of active reminders persisting in a patient’s EHR, the better the health care provided. The primary endpoint is a process outcome, i.e., the rate at which the issues reported by the reminders are resolved (resolution rates). Reminders will be considered resolved when the doctor acts to remove the cause that motivated the reminder. The primary analysis will consider all reminders, irrespective of disease. The CDSS will identify updates or changes in the patient status, and related reminders, by monitoring any changes in the patient EHR.

Secondary outcomes will explore resolution rates for reminders’ subgroup types as well as clinical outcomes. They are the resolution rates of (i) the reminders targeting primary care, (ii) the reminders targeting oncology care, (iii) the reminders targeting drugs that are the subjects of risk-sharing schemes, (iv) the reminders for venous thromboembolism (VTE) prevention, (v) in-hospital morbidity for VTE-related causes, (vi) VTE-related mortality, and (vii) in-hospital all causes mortality. VTE condition is a well-known problem in oncologic care, being a preventable yet still neglected disease. Research has shown the use of CDSSs to improve the assessment of patients’ risk for VTE, facilitate appropriate administration of prophylaxis interventions, and reduce the rate of symptomatic VTE in hospitalized patients [40–43]. We are interested in exploring the effectiveness of VTE reminders when they are combined together with several other types of reminders.

We will also explore any difference between the intervention and control groups regarding clinical characteristics, such as the site of tumor, physician, and inpatient/outpatient settings.

### Sample size

We calculated the sample size on the basis of the primary outcome. A previous systematic review assessing the effects of computer reminders delivered to clinicians at the point-of-care on healthcare processes and outcomes found a median improvement of 4.2 % in process adherence across all reported process outcomes [44]. Accordingly, assuming resolution rates of 5% in the intervention group versus 2 % in the control group (due to a possible group contamination), we calculated that a sample of 1306 reminders will be necessary to detect the difference between the two groups (power = 0.80; *α* = 0.05, two-sided; 1:1 allocation). Because estimates for intracluster correlation are not available, we increased the required sample size (by 10 %) to 1436 reminders to account for clustering by patient.

Moreover, based on a prior study evaluating EBMeDS, which recorded an average of 0.30 reminders per individuals triggered at baseline [45], we determined that a total number of approximately 4800 patients (2400 per group) need to be enrolled. This figure corresponds to a recruitment period of about 12 months for the oncology departments and the day hospital services of the IRST.

### Allocation and blinding

Anonymous patient identification (ID) numbers in the EHR system will be the unit of randomization. An individual external to the study group will generate the anonymous IDs using a formula based on patients’ unique fiscal code numbers.

We will randomly assign patients to either the control or experimental group with a 1:1 allocation. We will follow a computer-generated randomization schedule stratified by gender and age (0–30, 31–60, 61–80, and >80 years) using permuted blocks of random sizes [46]. Patients will be randomized immediately after the first launch of their EHR (entry of demographic data by physicians at hospital admission), and the allocation will be maintained through successive admissions.

Patients and study investigators (i.e., researchers, statisticians, information technology specialists, and hospital representatives) will be blinded to the allocation of participants. We will maintain the blinding up to the dataset disclosure. On the other hand, blinding of physicians is not feasible due to the nature of the intervention: the physician will know that a patient has been allocated to the intervention group if an automatic, patient-specific reminder is displayed on the screen.

### Data collection

The data collection for this study will follow the standard data collection procedures of the IRST. We will collect demographic (i.e., gender, age) and administrative (i.e., anonymous patient ID, admission and discharge dates, diagnoses the following data from the EHR archive on a regular basis) data from the EHR archive on a daily basis. Information on reminders, including all scripts that have been activated in a patient’s record, will also be collected daily, but during the night, so as not to disturb or slow down the use of the patient EHR.

### Statistical methods

For the primary outcome (i.e., resolution rates), the reminder will serve as the unit of analysis, and the patient the clustering factor. The patient will be the unit of analysis for the secondary outcomes (i.e., length of stay and in-hospital mortality). All analyses will follow the intention-to-treat principle: patients will be analyzed in the group to which they have been randomized. Descriptive statistics will be presented as means ± standard deviations (SD), medians and interquartile ranges (IQR), or percentages when appropriate. We will compare continuous variables using the Student’s *t* test when normally distributed, and the non-parametric two-sample Wilcoxon rank-sum (Mann-Whitney) test when they are not normally distributed. We will compare categorical variables using the chi-squared test or the Fisher’s exact test, as appropriate. To model the resolution rates of the reminders, we will run a random effects logistic regression analysis, accounting for clustering by patient [47].

For hypothesis testing, we will consider a probability level of less than 0.05 as statistically significant. All statistical tests will be two-sided. We will use the Stata software to perform all statistical analyses (Stata Corp., College Station, TX, USA).

### Data monitoring

Data monitoring will inform the ONCO-CODES trial conduct, identifying the potential need for adjustments:

(I) Sample size recalculation: Because the sample size calculation utilizes several assumptions, we will analyze the first batch of data collected and adjust the estimated sample size, if necessary, at the end of the sequential roll-out of the intervention. The 12-month recruitment period may also be adjusted, accordingly.
(II) Interim analysis: We will perform an interim analysis on the primary endpoint after 50 % of the patients have been randomized, after 50 % of the expected events have occurred, or after 6 months of the study’s initiation (the assumed half-life of the trial), whichever occurs first. An independent statistician that is blind to the patient allocation will perform the analysis. This analysis will inform whether the intervention has been proven for efficacy (beyond reasonable doubt). We will subsequently decide whether (or not) it is necessary to modify the study or prematurely terminate it, if necessary.
(III) End of trial: The end of trial will occur 30 days after the randomization of the last EHR. We will submit an end of trial notification and final report to the competent Ethical Committee, the IRST, and to the Sponsor.

### Harms

We do not anticipate any harms (or other unintended effects) to study participants. Intervention and control groups will differ in the presence (intervention) or absence (control) of automatic reminders displayed on physicians’ monitors. Patients assigned to the control group will receive usual care without the reminders. Nevertheless, we will consult an External Advisory Board in the event that the discontinuation of the study becomes an option due to unforeseeable reasons.

### Ethical and regulatory considerations

This study is conducted in accordance with the principles of the Declaration of Helsinki (October 2013) [48]. As the ONCO-CODES trial has a cluster design (several reminders, the unit of analysis, may derive from the same EHR, the unit of randomization), we followed the Ottawa statement to identify research participants and apply ethical and regulatory protections [49, 50]. The intervention (electronic CDSS reminders) does not directly target patients, but physicians who can be considered as the participants of the study. The risks associated with the participation of physicians in the ONCO-CODES trial are negligible. Physicians will be fully informed about the involvement of the IRST in the ONCO-CODES trial and trained to use the intervention. Requiring the signed consent of each physician is not feasible and will impact on the validity and generalizability of study results. Some have argued that healthcare professionals have an obligation to participate in health system or knowledge translation research [51, 52]. We consider that the waiver of signed consents will not adversely affect the rights or welfare of the research participants.

The Ethical Committee of IRST IRCCS Area Vasta Romagna approved the ONCO-CODES protocol.

### Protocol amendments

Any changes to the research protocol that may impact on the study conduct (e.g., changes in study design, eligibility criteria, study outcomes, sample size, study procedures, or significant administrative aspects) will require a formal amendment of the protocol. We will communicate any such amendments to the trial registry (ClinicalTrials.gov) and notify the health authorities in accordance with the Italian regulations. We will further seek the approval of the Ethical Committee for any amendments to the protocol.

### Confidentiality

The trial staff will ensure the maintenance of participants’ anonymity. The participants will be identified only by their initials and anonymous patient ID number. Depersonalized data will be extracted from the EHR. All documents will be stored securely and accessible only by the trial investigators and authorized personnel.

Clinical data collected during the study will only be accessible to the staff at IRST, thus complying with the current medical practice of the hospitals. The trial investigators external to the hospital (statistician, data manager, information technology personnel, etc.) will not access to any information at the patients level.

The ONCO-CODES trial will comply with the Italian Data Protection Act, which requires data to be anonymized as soon as it is practical to do so [53].

### Dissemination policy

The trial results will be posted on ClinicalTrial.gov as well as published in an open access medical journal.

We will further disseminate the study results to the health professionals of the IRST involved in the study. Datasets will be made available for research purposes upon request after the end of the study.

## Discussion

The results of our study will contribute to the current understanding of the effectiveness of CDSSs on cancer hospitals, thereby informing healthcare policy about the potential role of CDSS use in supporting integrated approaches between primary and specialty care. The increasing use of and familiarity with advanced technology among new generations of physicians call for pragmatic studies determining the optimal interface between primary and oncology care [54].

Another trial, named CODES trial (ClinicalTrials.gov: NCT02577198), is actually active [55]. It implements the same CDSS of the ONCO-CODES trial. However, its focus is on general hospitals and internal medicine patients. Therefore, it prioritizes an accurate implementation of the full spectrum of international reminders, covering multiple fields of knowledge and practice, rather than focusing on the impact of local and international reminders on a specific target population, i.e., cancer patients.

### Strengths and limitations

The ONCO-CODES trial has several strengths. First, in questions of effectiveness, the randomized controlled trial is the most appropriate research design. Second, we introduced a parallel qualitative study to elucidate why the CDSS does or does fit in clinical practice. The qualitative interviews will enable us to collect different attitudes and feedback, exploring the maturity of the technology. If the technology is not mature, it is likely that the results will be inconclusive and there will be no differences between the intervention and the control. It is always difficult to identify a right time to conduct a summative evaluation of a complex technology, but CDSSs seem to have more rather than less promise. So, the time is appropriate to pragmatically evaluate their effectiveness in large scale trials, accepting the risk that the technology under study has moved on by the time the trial is completed.

We preferred a pragmatic design under conditions that mimics the actual use of CDSSs in practice to increase the generalizability of the results as well as allows a more accurate estimation of the intervention’s true effectiveness. We must note the methodological limitation that physicians will not be blinded to the treatment allocation. When a patient-specific reminder is automatically displayed on the monitors, the physician will know that the particular patient belongs to the intervention group. We are aware that the unit of allocation (i.e., patient) and the lack of physician blinding can lead to possible group contamination: the doctors will be alternatively exposed to intervention and control group patients, applying the knowledge from a reminder generated for a patient allocated to the intervention group to a patient allocated to the control group. This possible learning effect (contamination of knowledge) can decrease the trial effect and lead to a more conservative effect estimate (i.e., towards the null). Randomization at the physician level, however, does not eliminate the possibility of contamination as physicians can care for patients across different wards; this level of randomization would, moreover, increase the organizational complexity of the study. A second factor that may decrease the trial effect is the adoption of an intention-to-treat analysis: not all physicians may adhere to the reminders within the study. Noncompliance is problematic because it results in a smaller difference between the intervention and comparison groups than may exists, thereby diluting the real impact of an intervention. Indeed, if the results will be positive, we will be confident that the CDSS is very likely to modify the resolution rates of the reminders, since most design factors work to dilute the effects.

### Conclusion

This is a pragmatic trial that is powered to find small (but important) effects of a CDSS on process outcomes. While a trial to assess true clinical endpoints would be ideal, the size and timeframe of such a trial would challenge its feasibility. Positive results in terms of improved adherence to reminders indicates that the CDSS intervention tested in this trial should help specialty physicians to close the gap between their intended and actual behaviors with respect to the primary and secondary care they provide for their patients. If the intervention works only in one setting (e.g., specialty care), it could be further tested trying more intensive approaches to facilitate interoperable EHR accessible to patients and health professionals at multiple facilities.

### Trial status

The implementation phase of the study was completed in October 2015, when the CDSS (MediDSS) was fully integrated with the hospital’s EHR (Cartella clinica oncologica). Subject recruitment and data collection began in November 2015 in the IRST hospital.

## Abbreviations

CDSS: Computerized decision support system
EBM: Evidence-based medicine
EBMeDS: Evidence-based medicine electronic decision support
EHR: Electronic health record
GFR: Glomerular filtration rate
IQR: Interquartile range
MediDSS: Medilogy Decision Support System
ONCO-CODES: COmputerized DEcision Support in ONCOlogy
RCT: Randomized controlled trial
SD: Standard deviation
VTE: Venous thromboembolism
